# The Role of Social Media in Shaping Decisions to Undergo Cosmetic Procedures: A Nationwide Cross-Sectional Study of the Syrian Population

**DOI:** 10.1093/asjof/ojaf103

**Published:** 2025-08-30

**Authors:** André Torbey, Perla Naji Yabroudi, Ahmad Al Darra, Saeed Kadri, Tarek Alsawaf, Jana Mugharbel, Julia Ammar Kheirbek, Sedra Al Habal, Hala Khair, Gretta Bachar Baghdan, Fares Kahal

## Abstract

**Background:**

Social media has become a major global force shaping beauty standards and aesthetic preferences. Although cosmetic procedures are increasingly normalized in Syria, the role of social media in influencing individuals’ decisions to pursue these interventions remains underexplored.

**Objectives:**

The aim of the authors of this study is to assess the influence of social media on attitudes and decision making regarding cosmetic procedures among the Syrian population, and to identify key demographic, psychological, and behavioral factors associated with this influence in the context of Syria's unique sociocultural and economic landscape.

**Methods:**

A cross-sectional study was conducted among 2605 participants using both online and paper-based questionnaires. The survey assessed demographics, attitudes toward cosmetic procedures, social media usage patterns, and the influence of influencers and digital content. Statistical analysis identified associations between digital exposure and procedural interest.

**Results:**

Participants spending >5 h daily on social media were significantly more likely to consider cosmetic procedures. Following a higher number of fashion influencers was strongly associated with appearance dissatisfaction and desire for cosmetic enhancements. Although 76% of participants accepted cosmetic procedures as socially valid, only 19% verified practitioner credentials, and 73% expressed distrust toward influencer promotions. Younger, female, and university-educated individuals were more susceptible to digital influence.

**Conclusions:**

Social media plays a substantial role in shaping cosmetic interests in Syria. These findings support the need for targeted strategies, including body image screening tools, media literacy programs, ethical advertising regulations, and transparent medical verification systems to promote informed and safe cosmetic decisions.

**Level of Evidence: 5 (Risk):**

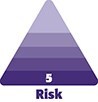

Globally, plastic surgery clinics once dedicated to reconstructive surgeries are now witnessing a significant shift in focus toward aesthetic enhancements, driven by the pursuit of “Instagram-perfect” beauty ideals.^1,2^ Cosmetic procedures have gained prominence as medical practices designed to enhance physical appearance and improve self-confidence. These include surgical interventions such as rhinoplasty and breast lifts, alongside nonsurgical treatments like dermal fillers and botulinum toxin injections, as outlined by the American Society of Plastic Surgeons.^3^ The demand for cosmetic procedures has surged significantly over the past decade throughout the world. According to a report by the International Society of Aesthetic Plastic Surgery in 2023, the total number of procedures performed worldwide increased by an impressive 40% over the 4 preceding years. Notably, even within a single year, from 2022 to 2023, the number of procedures grew by 3.4%.^4^ Understanding the factors that drive individuals to pursue cosmetic procedures is crucial in comprehending the growing demand for such interventions. Research conducted across various countries has identified a range of influencing factors, including age, gender, educational background, religion, self-esteem, socioeconomic status, and social media.^5-11^ The notable influence of social media has prompted modern surgeons to leverage platforms, such as Facebook and Instagram, to expand marketing outreach and showcase results of their procedures.^12^ Moreover, social media platforms offer a space for individuals to share their personal experiences with cosmetic procedures, both positive and negative, significantly influencing the perceptions and decisions of others. Recent studies from various countries highlight how social media not only shapes societal beauty standards but also acts as a powerful motivator for individuals considering cosmetic enhancements.^13-15^ Building on this, research suggests that increased time spent on social media intensifies its influence on individuals’ decisions to pursue cosmetic procedures.^16,17^ Although psychological variables were not the primary focus of this research, previous studies have highlighted their significant role in shaping individuals’ motivations toward cosmetic procedures. Furthermore, a study by Merino et al reviewed the impact of social media on self-esteem, mental health, and body image satisfaction, revealing how platforms like Instagram and Facebook exacerbate body dissatisfaction and psychological distress.^18^ This heightened dissatisfaction often leads individuals to seek cosmetic surgery as a solution to their perceived flaws. However, Jacobsen et al found that women who underwent cosmetic breast implantation had a higher risk of suicide compared with the general population.^19^ This underscores the importance of addressing the root causes of body image dissatisfaction through psychological support and public health interventions, rather than relying solely on cosmetic procedures, which can lead to more harm than good when performed for the wrong reasons. In a context like Syria, marked by diverse demographics, ideologies, and the strain of 14 years of war and economic instability, the influence of social media on body image raises intriguing questions. Despite the region's unique challenges, will Syrians mirror global trends, turning to cosmetic procedures driven by social media-induced beauty ideals? Or will their distinct circumstances shape a different response? Exploring these dynamics offers valuable insights into how social media's psychological and cultural impact transcends global and local boundaries. Although extensive research in developed countries, such as the United States, and neighboring nations, such as Saudi Arabia, has examined the influence of social media on cosmetic decision making, there is a notable lack of data exploring these dynamics within the Syrian context.^14,15^

This research investigates the influence of social media within the unique context of Syria. Through a nationwide cross-sectional study, it aims to assess the Syrian population's perspectives on cosmetic procedures, exploring the factors that influence their decisions to pursue or avoid such interventions, and more specifically; it examines the impact of social use on choices regarding both surgical and nonsurgical cosmetic enhancements, providing valuable insights into how global trends manifest in this distinct sociocultural and economic setting.

## METHODS

### Study Design and Population

This study employed a cross-sectional design to assess the influence of social media on cosmetic procedure decision making among the Syrian population. The study population comprised Syrians residing both within the country and abroad, with participants recruited through a convenience sampling strategy, which allowed for the broadest possible participation, ensuring a diverse representation of individuals across various demographic and socioeconomic backgrounds.

The study was conducted over a 3-and-a-half-month period, from September 1, 2024, to December 15, 2024. Inclusion criteria encompassed Syrian individuals aged 18 years or older who actively used social media and provided informed consent to participate. Individuals below the age of 18, non-Syrians, and those with incomplete responses were excluded. The required sample size was determined using the OpenEpi sample size calculator, based on an estimated Syrian adult population of 15 million.^20^ Using a 95% confidence level, a 5% margin of error, and an assumed proportion (*P*) of .5, the minimum sample size required was 385 participants. Following eligibility screening, 2605 participants were included in the final analysis, substantially exceeding the minimum requirement, thereby enhancing the statistical power and generalizability of the findings.

### Instrumentation and Data Collection

Data were collected using a self-administered questionnaire, developed following an extensive literature review and adapted from a validated instrument by Arab et al.^15^ To ensure its relevance and applicability to the Syrian population, modifications were made, and additional questions were incorporated. The questionnaire was designed in Arabic and underwent a forward-backward translation process, assisted by a professional translator, to maintain linguistic accuracy and conceptual equivalence. A pilot study involving 50 participants was subsequently conducted to assess the validity and reliability of the questionnaire. Beyond calculating a Cronbach's alpha score of 0.78, which indicated acceptable internal consistency, additional measures were taken to enhance validity. Content validity was ensured through expert review, where 3 independent specialists in plastic surgery, psychology, and social sciences evaluated the questionnaire for clarity, relevance, and cultural appropriateness. Face validity was assessed through participant feedback during the pilot phase, allowing for minor refinements to improve comprehension and response accuracy (Tables 1-6, Supplementary Tables 1-5).

**Table 1.** Attitudes Toward Advertisements for Cosmetic Procedures on Social Media

Do you prefer consulting a famous plastic surgeon on social media?	
Yes	859 (33)
No	1746 (67)
Do you think cosmetic surgery clinics appear more appealing if they were active on social media?	
Yes	1802 (69)
No	803 (31)
Have you ever inquired about a plastic surgeon's degrees and qualifications?	
Yes	494 (19)
No	2111 (81)
Do you agree with recording a cosmetic procedure with the patient's consent and using these videos for advertising purposes?	
Yes	511 (20)
Yes, but only for educational purposes	917 (35)
I don’t agree at all	1177 (45)
If you were to undergo cosmetic surgery, what reasons would you accept for allowing it to be filmed and shared on social media?	
For advertising purposes	206 (8)
Personal benefit (discount, offers, etc)	218 (8)
For educational purposes	615 (24)
I don’t agree to filming	1566 (60)
Do you support using social media to advertise cosmetic procedures?	
Yes, I support	1531 (59)
No, I don’t support	1074 (41)
What's the main benefit, in your opinion, of using social media to advertise surgeries and cosmetic procedures?	
Promoting cosmetic specialists and clinics	1908 (40)
Showing the results of cosmetic surgery	1338 (28)
Discounts and special offers	790 (16)
For educational purposes	791 (16)

**Table 2.** Demographic Characteristics of Participants

Variables	Influenced by social media's cosmetic advertisements		*P*-value
	Yes	No	
Age			<.0001*
Mean (±SD)	28.32 (±8.87)	30.09 (±10.82)	
Gender			<.0001*
Male	138 (19)	602 (81)	
Female	807 (43)	1058 (57)	
Educational level			.0123*
Illiterate	17 (28)	44 (72)	
Preparatory school	43 (28)	113 (72)	
High school	113 (34)	220 (66)	
University (medical education)	231 (37)	392 (63)	
University (nonmedical education)	475 (39)	743 (61)	
Master's degree	66 (31)	148 (69)	
Employment status			.249
Self-employment	131 (33)	262 (67)	
Student	300 (36)	542 (64)	
Working in a government job	313 (36)	550 (64)	
Unemployed	201 (40)	306 (60)	
Economic Level			.0191*
Low	50 (27)	134 (73)	
Moderate	262 (35)	495 (65)	
Good	463 (38)	764 (62)	
Very good	170 (39)	267 (61)	
Nationality			.0007*
Syrian in Syria	852 (36)	1531 (64)	
Syrian in the Middle East	57 (54)	49 (46)	
Syrian in Europe	20 (34)	39 (66)	
Syrian in other choices	5 (17)	24 (83)	
Syrian in the USA	11 (39)	17 (61)	

SD, standard deviation. *P < .05 was considered statistically significant.

**Table 3.** Participants’ Opinions and Experiences With Cosmetic Procedures

Variables	Influenced by social media's cosmetic advertisements		*P*-value
	Yes	No	
Do you believe cosmetic procedures are generally beneficial?			<.0001*
Yes	779 (46)	921 (54)	
No	166 (18)	739 (82)	
How familiar are you with cosmetic procedures?			<.0001*
No knowledge	44 (14)	281 (86)	
Basic knowledge	447 (35)	815 (65)	
Intermediate knowledge	338 (43)	454 (57)	
Advanced knowledge	116 (51)	110 (49)	
Cosmetic procedures are socially accepted			<.0001*
Yes	787 (40)	1193 (60)	
No	158 (25)	467 (75)	
Social media defines beauty standards			<.0001*
Yes	774 (43)	1022 (57)	
No	171 (21)	638 (79)	
Cosmetic procedures are exaggerated			.0089*
Yes	792 (37)	1322 (63)	
No	153 (31)	338 (69)	
Have you had any cosmetic surgical procedure?			<.0001*
Yes	244 (55)	203 (45)	
No	701 (32)	1457 (68)	
Have you had any aesthetic procedure?			<.0001*
Yes	382 (59)	263 (41)	
No	563 (29)	1397 (71)	
Are you interested in a cosmetic procedure?			<.0001*
Yes	720 (69)	330 (31)	
No	225 (14)	1330 (86)	

*P < .05 was considered statistically significant.

**Table 4.** Social Media Use and Exposure to Cosmetic Procedure Content

Variables	Influenced by social media's cosmetic advertisements		*P*-value
	Yes	No	
How many hours do you spend on social media			<.0001*
<2 h	138 (24)	447 (76)	
2-5 h	519 (37)	877 (63)	
>5 h	288 (46)	336 (54)	
Exposure to cosmetic procedure–related content			<.0001*
Minimal	179 (26)	515 (74)	
Significant	170 (50)	172 (50)	
Continuous	172 (53)	151 (47)	
Moderate	411 (42)	566 (58)	
No exposure	13 (5)	256 (95)	
Following plastic surgeons on social media			<.0001*
Yes	677 (55)	560 (45)	
No	268 (20)	1100 (80)	
Number of plastic surgeons followed			<.0001*
No one	268 (20)	1100 (80)	
From 1 to 5	474 (52)	437 (48)	
From 5 to 10	109 (60)	72 (40)	
>10	94 (65)	51 (35)	
Do you trust plastic surgeons on social media regarding cosmetic procedures			<.0001*
Yes	455 (58)	332 (42)	
No	490 (27)	1328 (73)	
Which doctor do you trust more			<.0001*
My personal doctor	575 (33)	1179 (67)	
I find no difference	226 (38)	442 (62)	
A doctor I follow on social media	104 (73)	39 (27)	
Following fashion influencers on social media			<.0001*
Yes	746 (45)	902 (55)	
No	199 (21)	758 (79)	
Number of fashion influencers followed			<.0001*
No one	199 (21)	758 (79)	
From 1 to 5	283 (36)	498 (64)	
From 5 to 10	188 (49)	195 (51)	
>10	275 (57)	209 (43)	
Do the fashion influencers you follow on social media promote cosmetic procedures			<.0001*
Yes	738 (45)	910 (55)	
No	207 (22)	750 (78)	
Do you trust fashion influencers regarding cosmetic procedures			<.0001*
Yes	311 (73)	114 (27)	
No	634 (29)	1546 (71)	

*P < .05 was considered statistically significant.

**Table 5.** Influence of Social Media on Body Image and Attitudes Toward Cosmetic Procedures

Variables	Influenced by social media's cosmetic advertisements		*P*-value
	Yes	No	
I constantly compare my appearance with social media celebrities			<.0001*
Yes	383 (71)	157 (29)	
No	562 (27)	1503 (73)	
I feel unattractive when I watch social media celebrities			<.0001*
Yes	315 (63)	187 (37)	
No	630 (30)	1473 (70)	
I would feel happier if my appearance resembled that of social media celebrities			<.0001*
Yes	360 (67)	180 (33)	
No	585 (28)	1480 (72)	
I might seriously consider undergoing a cosmetic procedure if it is popular among social media influencers.			<.0001*
Yes	306 (83)	62 (17)	
No	639 (28)	1598 (71)	
I might seriously consider undergoing a cosmetic procedure to enhance my image on social media			<.0001*
Yes	180 (76)	58 (24)	
No	765 (32)	1602 (68)	
I feel uncomfortable posting my photos on social media without using filters			<.0001*
Yes	309 (64)	174 (36)	
No	636 (30)	1486 (70)	
I constantly compare my photos on social media with those of others			<.0001*
Yes	303 (69)	136 (31)	
No	642 (30)	1524 (70)	
When I see other people's pictures, I feel pressure to change my look			<.0001*
Yes	277 (71)	114 (29)	
No	668 (30)	1546 (70)	
I might seriously consider getting cosmetic surgery if many of my friends do it			<.0001*
Yes	391 (76)	124 (24)	
No	554 (27)	1536 (73)	
I feel like society thinks a woman is “unattractive” if she doesn't get any cosmetic procedures			<.0001*
Yes	372 (49)	381 (51)	
No	573 (31)	1279 (69)	

*P < .05 was considered statistically significant.

**Table 6.** Logistic Regression of Factors Associated With Influencing by Social Media

Variables	Influenced by social media		*P*-value
	OR	95% CI	
How many hours do you spend on social media			
<2 h	Ref.		
2-5 h	1.27	0.99-1.63	.050
>5 h	1.77	1.33-2.35	<.0001*
Following fashion influencers on social media			.0140*
No	Ref.		
Yes	0.48	0.27-0.86	
Number of fashion influencers followed			
No one	Ref.		
From 1 to 5	2.94	1.91-4.51	<.0001*
From 5 to 10	4.69	2.40-9.15	<.0001*
>10	5.42	2.83-10.40	<.0001*
Gender			<.0001*
Male	Ref.		
Female	2.48	1.97-3.10	
Do you believe cosmetic procedures are generally beneficial?			<.0001*
No	Ref.		
Yes	3.02	2.45-3.73	
How familiar are you with cosmetic procedures?			
No knowledge	Ref.		
Basic knowledge	1.80	1.24-2.60	.0017*
Intermediate knowledge	2.20	1.50-3.21	<.0001*
Advanced knowledge	2.82	1.79-4.45	<.0001*
Cosmetic procedures define beauty standards			<.0001*
No	Ref.		
Yes	2.51	2.03-3.10	

OR, odds ratio. *P < .05 was considered statistically significant.

The final questionnaire consisted of 53 questions structured into 6 main sections, covering demographic information, opinions on plastic and reconstructive surgery, personal experience with cosmetic procedures, general social media usage, social and psychological effects of social media, and opinions on cosmetic surgery advertising on social media. The demographic section collected data on age, gender, educational level, employment status, income, nationality, and residency. The second section examined perceptions of plastic and reconstructive surgery, its accessibility, and societal attitudes. The third section explored personal and familial experiences with cosmetic procedures, including motivations and deterrents. The fourth section assessed social media engagement, preferred platforms, sources of information on cosmetic procedures, and interactions with influencers and medical professionals. The fifth section focused on the psychological and social effects of social media, addressing its role in shaping body image, self-esteem, and emotional well-being. The final section evaluated opinions on cosmetic surgery advertising, including trust in influencer-endorsed content and concerns regarding the ethics of such promotions.

The questionnaire was distributed through both online and paper-based formats. The online survey, hosted on Google Forms, was disseminated via Facebook, Instagram, and WhatsApp groups. The study information was clearly outlined at the top of the form, specifying that completion and submission of the questionnaire constituted informed consent, ensuring that participants were fully aware of the research objectives and data confidentiality measures. No monetary incentives or promotional advertisements were used for recruitment. To ensure broad accessibility, paper-based surveys were distributed in universities, hospitals, private clinics, restaurants, cafés, and public institutions. These surveys were handed out randomly to individuals willing to participate, collected on the spot, and later digitized to ensure consistency in data management. Participants were provided with a brief explanation of the study, and verbal informed consent was obtained before completion. Responses with missing data were excluded during the data cleaning process to maintain data integrity.

### Statistical Analysis

The statistical analysis in the study was conducted using SPSS version 28 and incorporated a range of methods to interpret the data and derive meaningful insights. Initially, descriptive statistics, including means, standard deviations, and frequencies, were applied to summarize the main characteristics of the dataset. For inferential analysis, *t*-tests and χ^2^ tests were employed to assess statistical significance. Additionally, multiple logistic regression was used in the multivariate analysis to calculate adjusted odds ratios (ORs), accounting for potential confounding variables.

### Ethical Considerations

Ethical approval for the study was granted by the IRB of the Syrian Private University (serial number: PH-24112024-82). The study adhered to established ethical research guidelines, ensuring that participants were fully informed about the study objectives, data confidentiality measures, and voluntary participation rights. All responses were collected anonymously, and no personally identifiable data were stored. Paper-based responses were securely stored before digitization, and all electronic data were maintained in a password-protected database, accessible only to the research team. The study aligns with the Declaration of Helsinki.

## RESULTS

A total of 2605 participants were included in the study, with a mean age of 29.45 ± 10.19 years. The majority of respondents were female (72%), and nearly half (47%) reported a “good” family monthly income. The vast majority (91%) were residing in Syria, with a significant proportion (69%) located in Damascus.

**Figure 1.**
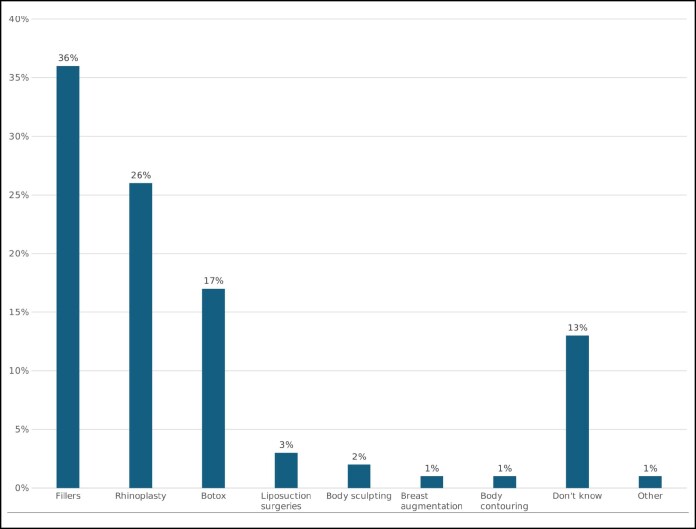
Most exaggerated procedure.

Overall, 65% of participants considered cosmetic procedures beneficial, and 80% believed they were appropriate for both genders. When assessing knowledge levels, 48% of respondents reported only basic knowledge, whereas 30% had intermediate knowledge. A substantial majority (76%) recognized that cosmetic procedures are socially accepted, yet 81% believed they were excessively performed. Fillers (36%) and rhinoplasty (26%) were the most frequently cited overused procedures, followed by Botox (17%; Figure 1). Notably, 69% of participants acknowledged that social media significantly contributes to shaping modern beauty standards.

**Figure 2.**
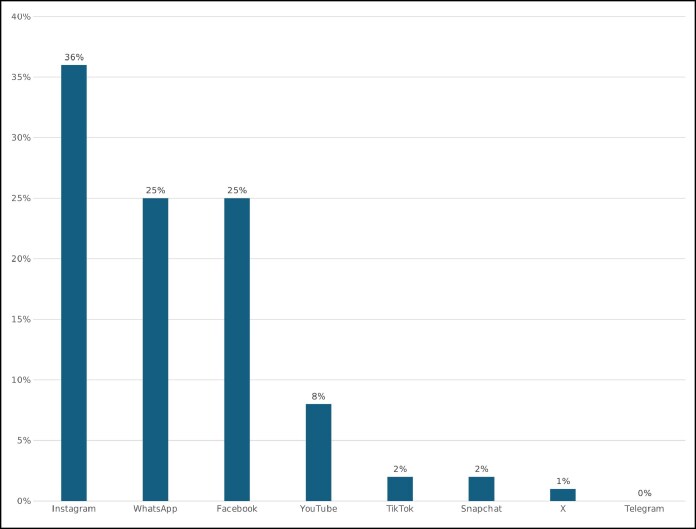
The most commonly used social media applications among participants.

Among participants, 36% (*n* = 945) reported being influenced by social media advertisements to consider undergoing cosmetic procedures. The majority (55%) expressed interest in nonsurgical treatments, whereas 16% considered surgical procedures, and 29% showed interest in both. Notably, 76% of those influenced by advertisements (*n* = 720) intended to undergo a cosmetic procedure in the future. Additionally, 42% (*n* = 1092) had already undergone at least 1 cosmetic procedure. Social influence played a critical role, with 75% of participants reporting that they had friends who had undergone cosmetic procedures, and 49% indicating that at least 1 family member had undergone a cosmetic intervention. Self-satisfaction was cited as the primary motivation for undergoing cosmetic procedures (43%), whereas lack of perceived need (26%) was the most frequently cited reason for avoidance. The majority of respondents (78%) reported spending between 2 and >5 h daily on social media, with Instagram emerging as the most frequently used platform (Figure 2). Regarding sources of information on cosmetic procedures, 37% of participants relied on medical professionals, whereas 22% turned to online search engines such as Google. Interestingly, 63% of respondents followed fashion influencers, whereas a smaller proportion followed plastic surgeons. Additionally, 63% of participants noted that influencers actively endorse cosmetic procedures. Those who followed plastic surgeons cited their motivation as gaining insights into future treatments and educational purposes. Despite nearly half of the participants following plastic surgeons on social media, only 33% expressed a preference for consulting a plastic surgeon with an active social media presence, and 19% reported actively verifying the credentials of such surgeons. Although 69% believed that clinics with a social media presence appeared more credible, only 35% supported the filming of cosmetic procedures for educational purposes, and merely 8% expressed willingness to allow their own procedures to be filmed and used publicly. Regarding the function of social media in promoting cosmetic surgery, 40% perceived it primarily as a marketing tool used by specialists and clinics. A notable proportion of participants reported experiencing negative psychological effects due to social media exposure. Twenty-four percent frequently compared themselves with influencers, whereas 19% reported feeling unattractive as a result of exposure to online beauty standards. However, only 21% believed they would be happier if they resembled social media celebrities. Additionally, 14% stated that they would consider cosmetic procedures solely based on influencer promotions, and 15% felt peer pressure to enhance their appearance for social media engagement. Despite these pressures, the majority of participants rejected the notion that women who do not undergo cosmetic procedures are considered less attractive by society. Statistical analysis revealed significant associations between social media influence and several demographic and behavioral variables (*P* < .0001). Younger participants (mean age 28.32 years), females (43%), and university-educated individuals (medical students: 37%, nonmedical students: 39%) were more likely to report being influenced by social media in their cosmetic procedure decision making. The probability of considering a cosmetic procedure was notably higher among those with continuous exposure to cosmetic content (53%), those who followed plastic surgeons (55%), and those who engaged with fashion influencers (45%). Social comparison emerged as a strong determinant, with 71% of participants feeling pressure to alter their appearance, and 76% stating they would be more likely to consider a cosmetic procedure if their friends had undergone one. However, skepticism toward social media influencers was prevalent, as 73% of participants expressed distrust toward influencers promoting cosmetic procedures, and 69% rejected the notion that social media dictates attractiveness standards for women. Notably, individuals who spent >5 h daily on social media were significantly more likely to express an interest in undergoing cosmetic treatments (46%).

Multivariate logistic regression identified key predictors of social media influence on cosmetic decision making. Individuals who spent >5 h daily on social media were at significantly higher risk of being influenced (OR = 1.77, *P* < .0001). Following >10 fashion influencers increased the likelihood of being influenced by 5.42 times (*P* < .0001). Women were 2.48 times more likely than men to report social media influence on their cosmetic decisions (*P* < .0001).

Additional predictors included positive perceptions of cosmetic procedures (OR = 3.02, *P* < .0001), advanced knowledge of aesthetic interventions (OR = 2.82, *P* < .0001), and the belief that cosmetic procedures define modern beauty standards (OR = 2.51, *P* < .0001; Figures 1, 2).

## DISCUSSION

Social media has evolved beyond passive consumption, shaping self-perception, decision making, and mental health while influencing modern beauty standards.^15,21^ This has contributed to a growing sense of body dissatisfaction and an unconscious drive to alter one's appearance to align with digitally propagated ideals.^22^ Although the influence of social media has been widely examined in other regions, its multifaceted effects in Syria—a country shaped by prolonged conflict, shifting cultural norms, and economic instability—remain largely unexamined.^23^ This study fills that gap by exploring how peer pressure, digital exposure, and influencer marketing correlate with cosmetic preferences and procedural intent within this unique setting. It contributes to broader conversations around the globalization of aesthetic values, the ethics of online beauty marketing, and the psychological implications of appearance-based comparison.

Involving 2605 participants, our study found that the average age among those influenced by social media cosmetic content was 28.32 years. The majority were female (72%), which suggests that, in Syrian society, women may face more pronounced aesthetic expectations, mirroring global and regional trends where female appearance remains a more scrutinized domain.^24,25^ Additionally, male underreporting due to stigma could have skewed these proportions. Educational level and economic status were significantly associated with cosmetic interest. Participants with university degrees (74.7%) and higher income levels (38.9%) were more likely to report being influenced by social media. These results align with findings from China, Iran, and the United States, which associate higher education and socioeconomic status with greater engagement in cosmetic enhancement.^26-28^ The reasons may include improved access to information, heightened aesthetic awareness, and self-imposed beauty standards shaped by social exposure.^10,29^

Our findings show a high prevalence of aesthetic interventions—55% of participants reported having undergone surgical cosmetic procedures, and 59% had nonsurgical treatments. Notably, 65% viewed cosmetic procedures positively, and 76% considered them socially acceptable. These findings echo studies in Saudi Arabia and the United States where generational shifts and body image culture contribute to growing procedural normalization.^30,31^ In Syria, the normalization may be further influenced by lower costs and postconflict recovery narratives, where individuals perceive physical transformation as a means of empowerment.^32,33^ Higher self-reported knowledge of cosmetic procedures was correlated with increased likelihood of being influenced, suggesting that online information—especially on social platforms—may simultaneously educate and promote aspirational beauty ideals. Previous work by Montemurro et al reported that over 95% of cosmetic patients consulted online sources before undergoing procedures, underscoring the importance of digital content in shaping procedural interest.^34^ Participants with daily social media usage exceeding 5 h demonstrated a significantly higher likelihood of considering cosmetic procedures. This aligns with previous studies in Saudi Arabia, reinforcing the theory that prolonged exposure to curated content is associated with greater body dissatisfaction and interest in aesthetic enhancements.^15,35^ Social Comparison Theory helps explain these patterns: individuals assess their own appearance in relation to idealized figures such as influencers, which may lead to feelings of inadequacy.^36^ Our data also revealed that 76% of participants believed undergoing procedures could help them resemble social media celebrities, with 67% thinking this would increase happiness. Additionally, 63% followed fashion influencers who frequently promote cosmetic interventions, and the number of influencers followed was positively associated with procedural interest. The concept of Fear of Missing Out may further contribute to internalizing beauty standards and the perceived need to “keep up” with digital trends.^37^ Despite active engagement with influencer content, 73% of participants expressed distrust toward influencers who promote cosmetic procedures. Furthermore, although many followed plastic surgeons, only 19% verified their credentials. This contradiction highlights a need for ethical promotion and greater transparency in digital health communication Our findings revealed that a significant subset of participants pursued behaviors that may be associated with body image distress, which include persistent appearance comparisons (71%), discomfort with unfiltered self-images (64%), and a belief that cosmetic procedures could increase happiness (67%). These tendencies, while not directly evaluated through diagnostic methods, it may indicate a psychological profile that may benefit from structured assessment. In this context, it may be beneficial for future studies to involve instruments like Body Dysmorphic Disorder Questionnaire (BDDQ) or its aesthetic variant (BDDQ-AS) which have demonstrated high sensitivity and reasonable specificity in detecting body dysmorphic tendencies.^38-40^ Such preventive evaluation can potentially assist differentiating between healthy aesthetic motivations and those resulting from unfulfilled psychological demands, eventually guiding safer and more ethical procedures.

Our findings also support the development of national media literacy initiatives targeted at adolescents and young adults. These programs—ideally implemented through schools and digital public health campaigns—should teach users to critically assess curated content, recognize image manipulation, and understand the emotional consequences of appearance-based comparisons. Promoting digital resilience can empower individuals to engage with cosmetic choices from a place of informed self-awareness, rather than external pressure.^41^ The study's findings emphasize the need for more proactive frameworks that go beyond traditional education and regulation. Health professionals should explore developing standardized preconsultation processes that incorporate not just medical risk disclosure but also visual expectation management, which addresses the impact of digitally changed pictures. Clinics should implement consultation models that include social media usage as part of the patient's history, acknowledging its significance in influencing procedural intent. Furthermore, collaborations between medical associations and social media platforms may enable the labeling of promotional content from recognized practitioners, allowing people to differentiate between evidence-based messages and commercially motivated influence.^42^ These strategies together contribute to a more transparent, psychologically aware, and ethically grounded aesthetic environment.

### Limitations

This study offers valuable insight into the influence of social media on cosmetic decision making in Syria; however, several limitations must be acknowledged. First, the cross-sectional design limits our ability to establish causality between digital exposure and procedural intent. Second, although the questionnaire was distributed both online and via paper-based forms to increase accessibility, the primary reliance on social media platforms for data collection introduces inherent selection bias. This may have led to an overrepresentation of younger, digitally engaged, and urban participants, potentially limiting the generalizability of our findings to the broader Syrian population. Third, self-reported data on cosmetic procedures and psychological influences may be affected by social desirability bias or memory recall limitations. Additionally, although this study explores key associations such as influencer impact and screen time, it does not include in-depth psychological assessments such as validated BDD diagnoses, nor does it explore long-term post-procedure satisfaction or mental health outcomes.

## CONCLUSIONS

This study presents one of the first large-scale investigations into the relationship between social media and cosmetic procedure decisions in Syria. Drawing on responses from 2605 participants, it reveals a population increasingly influenced by digital beauty norms, yet discerning and cautious in their aesthetic choices. A key finding was the strong correlation between excessive social media use—particularly over 5 h daily—and increased likelihood of being influenced to undergo cosmetic procedures. The cumulative effect of influencer exposure, comparison-driven dissatisfaction, and algorithmically tailored content appears to play a significant role in shaping procedural intent.

At the same time, the majority of participants expressed skepticism toward influencers and rarely verified the credentials of aesthetic providers. This contradiction reflects a growing aesthetic desire amid a fragmented informational landscape. These findings underscore the urgency of implementing screening tools like the BDDQ, expanding media literacy education, enforcing transparent marketing standards, and establishing verifiable practitioner registries. Such systems are essential for bridging the gap between interest and safety—particularly in a postconflict setting where identity, agency, and appearance often intersect.

## Supplementary Material

ojaf103_Supplementary_Data
