# Supplementary material for: The Role of Social Media in Shaping Decisions to Undergo Cosmetic Procedures: A Nationwide Cross-Sectional Study of the Syrian Population
Source: Aesthet Surg J Open Forum. 2025 Aug 30;7:ojaf103. doi: 10.1093/asjof/ojaf103 (PMC12464937; doi:10.1093/asjof/ojaf103)
Supplement: ojaf103_Supplementary_Data [file ojaf103_supplementary_data.docx]

- **Demographics:**

The survey included 2605 participants, with a mean age of 29.45 ± 10.19 years. The majority were female (72%), predominantly from non-medical educational backgrounds (47%), and 47% reported a family monthly income classified as good. Most respondents were Syrian residents (91%), with 69% residing in Damascus. (Table 1)

**Table 1: Participants’ characteristics.**

| **Age** |  |
| --- | --- |
| Mean (±SD) | 29.45 (±10.19) |
| Lowest value | 18 |
| Highest value | 76 |
| **Gender** | **N** (%) |
| Male | 740 (28) |
| Female | 1865 (72) |
| **Educational level** |  |
| Illiterate | 61 (2) |
| Preparatory school | 156 (6) |
| High school | 333 (13) |
| University (medical education) | 623 (24) |
| University (non-medical education) | 1218 (47) |
| Master’s degree | 214 (8) |
| **Employment Status** |  |
| Self-employment | 393 (15) |
| Student | 842 (32) |
| Working in a government job | 863 (33) |
| Unemployed | 507 (19) |
| **Economic Level** |  |
| Low | 184 (7) |
| Moderate | 757 (29) |
| Good | 1227 (47) |
| Very good | 437 (17) |
| **Nationality** |  |
| Syrian in Syria | 2383 (91) |
| Syrian in the Middle East | 106 (4) |
| Syrian in EUROPE | 59 (2) |
| Syrian in other choices | 29 (1) |
| Syrian in the U.S.A | 28 (1) |
| **Residency** |  |
| Damascus | 1786 (69) |
| Another province | 597 (23) |
| Not in Syria | 222 (8) |

- **General Knowledge about cosmetic procedure**

Among the participants, 65% considered cosmetic procedures generally beneficial, with 80% believing they are suitable for both genders. While 76% viewed these procedures as socially accepted, 81% perceived them as exaggerated. Additionally, 69% felt that social media define beauty standards. Familiarity levels varied, with 48% having basic knowledge and 30% intermediate knowledge. Fillers (36%) and rhinoplasty (26%) were identified as the most exaggerated procedures, followed by Botox (17%). (Table 2)

**Table 2: General Knowledge About Cosmetic Procedure.**

| **Do you believe cosmetic procedures are generally beneficial?** |  |
| --- | --- |
| Yes | 1700 (65) |
| No | 905 (35) |
| **How familiar are you with cosmetic procedures?** |  |
| No knowledge | 325 (12) |
| Basic knowledge | 1262 (48) |
| Intermediate knowledge | 792 (30) |
| Advanced knowledge | 226 (9) |
| **Cosmetic procedures are for** |  |
| Males | 3 (0) |
| Females | 507 (19) |
| Both | 2095 (80) |
| **Cosmetic procedures are socially accepted** |  |
| Yes | 1980 (76) |
| No | 625 (24) |
| **Social media defines beauty standards** |  |
| Yes | 1796 (69) |
| No | 809 (31) |
| **Cosmetic procedures are exaggerated** |  |
| Yes | 2114 (81) |
| No | 491 (19) |
| **Most exaggerated procedure** |  |
| Fillers | 1230 (36) |
| Rhinoplasty | 890 (26) |
| Botox | 601 (17) |
| Liposuction surgeries | 89 (3) |
| Body sculpting | 80 (2) |
| Other cosmetic procedure | 40 (1) |
| Breast augmentation | 38 (1) |
| Body contouring | 24 (1) |
| Don’t know | 456 (13) |

- **Influence of Social Media on the Decision to Undergo Cosmetic Procedures**

The study found that 36% of respondents (n = 945) were influenced by advertisements to consider cosmetic treatments, with 55% favoring nonsurgical options, 16% considering surgical procedures, and 29% interested in both. Among those influenced by social media ads, 76% (n = 720) planned future treatments, and 42% (n = 1092) had already undergone cosmetic procedures. Additionally, 75% had friends who had undergone such treatments, compared to 49% with family members. Self-satisfaction (43%) is the primary motivation for cosmetic procedures, while lack of need (26%) is the main deterrent. (Table 3)

**Table 3: Influence of Social Media on the Decision to Undergo Cosmetic Procedures**

| **Have you had any cosmetic surgical procedure?** |  |
| --- | --- |
| Yes | 447 (17) |
| No | 2158 (83) |
| **Have you had any aesthetic procedure?** |  |
| Yes | 645 (25) |
| No | 1960 (75) |
| **Has any member of your family had a cosmetic procedure?** |  |
| Yes | 1274 (49) |
| No | 1331 (51) |
| **Has any of your friends had a cosmetic procedure** |  |
| Yes | 1946 (75) |
| No | 659 (25) |
| **Influenced by social media’s cosmetic advertisements** |  |
| Yes | 945 (36) |
| No | 1660 (64) |
| **Are you interested in a cosmetic procedure?** |  |
| Yes | 720 (76) |
| No | 225(24) |
| **If cosmetic treatment advertisements on social media influenced you, which type of cosmetic treatment are you considering undergoing?** |  |
| Surgical procedure | 151 (16) |
| Non-surgical procedure | 516 (55) |
| Both | 278 (29) |
| **If you're interested, what motivates your interest?** |  |
| Self-satisfaction | 1513 (43) |
| Medical advice | 731 (21) |
| Social media | 386 (11) |
| I don’t want | 325 (9) |
| Society pressure | 189 (5) |
| Pressure from partner | 120 (3) |
| Better treatment from the others | 115 (3) |
| Professional development | 100 (3) |
| Everybody is doing it and I’ll feel guilty if I didn’t | 80 (2) |
| **What stops you from doing a cosmetic procedure** |  |
| I don’t need | 940 (26) |
| Nothing | 662 (18) |
| Cost | 622 (17) |
| Religious | 468 (13) |
| I don’t trust plastic surgeons | 325 (9) |
| Fear of addiction | 252 (7) |
| Bad experience with relatives | 248 (7) |
| Society pressure | 161 (4) |

- **General Use of Social Media**

The findings show that 78% of participants spent 2–5+ hours daily on social media, with Instagram being the most popular platform (Figure 2). While 37% sought cosmetic procedure information from doctors, 22% used websites like Google. Fashion influencers had more followers (63%) than plastic surgeons, with 63% of viewers noting they endorse cosmetic procedures. Participants followed plastic surgeons mainly for future treatment information and educational insights. (Table 4)

**Table 4: General Use of Social Media**

| **How many hours do you spend on social media** |  |
| --- | --- |
| Less than 2 hours | 585 (22) |
| 2-5 hours | 1396 (54) |
| More than 5 hours | 624 (24) |
| **Which social media app do you use the most** |  |
| Instagram | 1808 (36) |
| WhatsApp | 1261 (25) |
| Facebook | 1248 (25) |
| YouTube | 400 (8) |
| TikTok | 97 (2) |
| Snapchat | 91 (2) |
| X | 46 (1) |
| Telegram | 19 (0.3) |
| **Source of information** |  |
| Doctor | 1625 (37) |
| Google and internet websites | 979 (22) |
| Friends and relatives | 645 (15) |
| Social media | 617 (14) |
| Cultural seminars | 297 (7) |
| T.V program | 137 (3) |
| Journal | 77 (2) |
| **Exposure to cosmetic procedures-related content** |  |
| Minimal | 694 (27) |
| Significant | 342 (13) |
| Continuous | 323 (12) |
| Moderate | 977 (38) |
| No exposure | 269 (10) |
| **Following plastic surgeons on social media** |  |
| Yes | 1237 (47) |
| No | 1368 (53) |
| **Number of plastic surgeons followed** |  |
| No one | 1368 (53) |
| From 1 to 5 | 911 (35) |
| From 5 to 10 | 181 (7) |
| More than 10 | 145 (6) |
| **Reason for following a plastic surgeon on social media** |  |
| I don’t follow any | 1368 (53) |
| For entertainment only | 375 (14) |
| For educational or professional purposes | 406 (16) |
| Gathering information | 456 (17) |
| **Do you trust plastic surgeons on social media regarding cosmetic procedures** |  |
| Yes | 787 (30) |
| No | 1818 (70) |
| **Which doctor do you trust more** |  |
| My personal doctor | 1754 (67) |
| I find no difference | 708 (27) |
| A doctor I follow on social media | 143 (5) |
| **Following fashion influencers on social media** |  |
| Yes | 1648 (63) |
| No | 957 (37) |
| **Number of fashion influencers followed** |  |
| No one | 957 (37) |
| From 1 to 5 | 781 (30) |
| From 5 to 10 | 383 (15) |
| More than 10 | 484 (19) |
| **Do the fashion influencers you follow on social media promote cosmetic procedures** |  |
| Yes | 1632 (63) |
| No | 973 (37) |
| **Do you trust fashion influencers regarding cosmetic procedures** |  |
| Yes | 425 (16) |
| No | 2180 (84) |

- **Psychosocial Effects of Viewing Cosmetic-Related Material on Social Media**

Nearly a quarter of respondents often compared themselves to social media influencers, with 19% feeling unattractive and only 21% believing they’d be happier resembling them. Just 14% would consider cosmetic procedures popularized by influencers, and 15% felt pressured by peers to enhance their appearance for social media. Most participants rejected the notion that society views women as “unattractive” for not undergoing cosmetic treatments. (Table 5)

**Table 5: Psychosocial Effects of Viewing Cosmetic-Related Material on Social Media**

| **I constantly compare my appearance with social media celebrities** |  |
| --- | --- |
| Yes | 540 (21) |
| No | 2065 (79) |
| **I feel unattractive when I watch social media celebrities** |  |
| Yes | 502 (19) |
| No | 2103 (81) |
| **I would feel happier if my appearance resembled that of social media celebrities** |  |
| Yes | 540 (21) |
| No | 2065 (79) |
| **I might seriously consider undergoing a cosmetic procedure if it is popular among social media influencers.** |  |
| Yes | 368 (14) |
| No | 2237 (86) |
| **I might seriously consider undergoing a cosmetic procedure to enhance my image on social media.** |  |
| Yes | 238 (9) |
| No | 2367 (91) |
| **I feel uncomfortable posting my photos on social media without using filters** |  |
| Yes | 483 (18) |
| No | 2122 (81) |
| **I constantly compare my photos on social media with those of others** |  |
| Yes | 439 (17) |
| No | 2166 (83) |
| **When I see other people's pictures, I feel pressure to change my look** |  |
| Yes | 391 (15) |
| No | 2214 (85) |
| **I might seriously consider getting cosmetic surgery if many of my friends do it** |  |
| Yes | 515 (20) |
| No | 2090 (80) |
| **I feel like society thinks a woman is 'unattractive' if she doesn't get any cosmetic procedures** |  |
| Yes | 743 (29) |
| No | 1862 (71) |
